# Random Forest Algorithm for the Classification of Neuroimaging Data in Alzheimer's Disease: A Systematic Review

**DOI:** 10.3389/fnagi.2017.00329

**Published:** 2017-10-06

**Authors:** Alessia Sarica, Antonio Cerasa, Aldo Quattrone

**Affiliations:** ^1^Institute of Bioimaging and Molecular Physiology, National Research Council, Catanzaro, Italy; ^2^Institute of Neurology, University Magna Graecia, Catanzaro, Italy

**Keywords:** random forest, Alzheimer's disease, mild cognitive impairment, neuroimaging, classification

## Abstract

**Objective:** Machine learning classification has been the most important computational development in the last years to satisfy the primary need of clinicians for automatic early diagnosis and prognosis. Nowadays, Random Forest (RF) algorithm has been successfully applied for reducing high dimensional and multi-source data in many scientific realms. Our aim was to explore the state of the art of the application of RF on single and multi-modal neuroimaging data for the prediction of Alzheimer's disease.

**Methods:** A systematic review following PRISMA guidelines was conducted on this field of study. In particular, we constructed an advanced query using boolean operators as follows: *(“random forest” OR “random forests”) AND neuroimaging AND (“alzheimer's disease” OR alzheimer's OR alzheimer) AND (prediction OR classification)*. The query was then searched in four well-known scientific databases: Pubmed, Scopus, Google Scholar and Web of Science.

**Results:** Twelve articles—published between the 2007 and 2017—have been included in this systematic review after a quantitative and qualitative selection. The lesson learnt from these works suggest that when RF was applied on multi-modal data for prediction of Alzheimer's disease (AD) conversion from the Mild Cognitive Impairment (MCI), it produces one of the best accuracies to date. Moreover, the RF has important advantages in terms of robustness to overfitting, ability to handle highly non-linear data, stability in the presence of outliers and opportunity for efficient parallel processing mainly when applied on multi-modality neuroimaging data, such as, MRI morphometric, diffusion tensor imaging, and PET images.

**Conclusions:** We discussed the strengths of RF, considering also possible limitations and by encouraging further studies on the comparisons of this algorithm with other commonly used classification approaches, particularly in the early prediction of the progression from MCI to AD.

## Introduction

The Alzheimer's disease (AD), a common form of dementia, is a progressive neurodegenerative disorder that affects mostly elderly people (Berchtold and Cotman, 1998). It is characterized by a decline in cognitive function, including progressive loss of memory, reasoning, and language (Collie and Maruff, 2000). Mild cognitive impairment (MCI) is an intermediate state between healthy aging and AD, which is not severe enough to interfere with daily life. Although not all MCI subjects develop to AD and they remain cognitively stable for many years, the incidence of progression is evaluated between 10 and 15% per year (Palmqvist et al., 2012). There is no generally accepted cure for AD, but several treatments exist for delaying its course. For this reason, it is extremely important to early detect the MCI subjects that are at imminent risk of conversion to AD.

The diagnosis of AD is based primarily on multiple variables and factors, such as, demographics and genetic information, neuropsychological tests, cerebrospinal fluid (CSF) biomarkers, and brain imaging data. Moreover, for the assessment of the risk of conversion from MCI, the rate of change of these variables could represent a further source of knowledge. In particular, the neuroimaging technologies, such as, magnetic resonance imaging (MRI), functional MRI (fMRI), diffusion tensor imaging (DTI), single photon emission tomography (SPECT), and positron emission tomography (PET) have been widely and successfully applied in the study of MCI and AD (Greicius et al., 2004; Matsuda, 2007; Fripp et al., 2008; Frisoni et al., 2010; Acosta-Cabronero and Nestor, 2014). The choice of the neuroimaging modality depends on the duration and severity of the disease, for example when MRI could not reveal any brain alterations, fMRI, SPECT, or PET are able to assess metabolic abnormalities and DTI could be used for investigating the microstructural disruption of the white matter (WM).

The high dimension of all the features considered in the diagnosis of AD and in the progression from MCI, and their complex interactions make it very difficult for humans to interpret the data. Computer aided diagnosis (CAD) represents a valuable automatic tool for supporting the clinicians by teaching to computers to predict incipient AD. Machine learning and pattern recognition algorithms have been proven to efficiently classify AD patients and healthy controls (HC) and to distinguish between stable MCI (sMCI) subjects and progressive MCI (pMCI) that converted to AD (Zhang et al., 2012; Falahati et al., 2014; Trzepacz et al., 2014). In general, the machine learning methods used on neuroimaging data rely on a single classifier, such as, the widely used Support Vector Machine (SVM), Linear Discriminant Analysis (LDA), or Naïve Bayes. However, in the last years, ensembles algorithms resulted to be a reliable alternative to single classifiers showing better performance than the latter, especially when multi-modality variables are combined together. Although among all ensembles approaches Random Forest (RF) (Breiman, 2001) produced the best accuracies in many scientific fields (Menze et al., 2009; Calle et al., 2011; Chen et al., 2011) and in other neurological diseases (Sarica et al., 2017), it is still poorly applied in the prediction of AD, and only lately researchers payed their attention to it. In particular, RF showed important advantages over other methodologies regarding the ability to handle highly non-linearly correlated data, robustness to noise, tuning simplicity, and opportunity for efficient parallel processing (Caruana and Niculescu-Mizil, 2006). Moreover, RF presents another important characteristic: an intrinsic feature selection step, applied prior to the classification task, to reduce the variables space by giving an importance value to each feature.

For all these reasons, the main goal of this systematic review was to highlight the role of RF as the ideal candidate for handling the high-dimensional problem and the variable redundancy in the early diagnosis of AD. We sought to review the literature in this area to identify all the works that applied the RF algorithm on single and multi-modality neuroimaging data, eventually combined with demographics and genetic information, and with neuropsychological scores. Our aim was also to evaluate how well, in term of accuracy, RF was able to classify AD and to distinguish between sMCI and pMCI, and how its intrinsic feature selection procedure could improve this overall accuracy.

### Random forest algorithm

**Figure 1:**
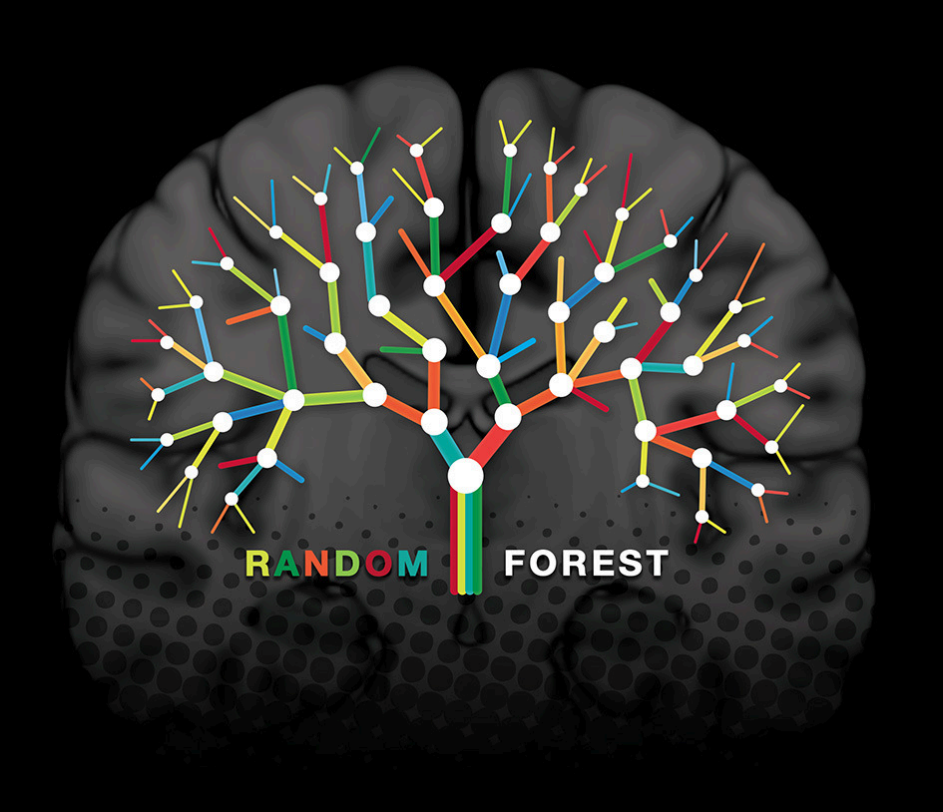
Illustration of a random forest construct superimposed on a coronal slice of the MNI 152 (Montreal Neurological Institute) standard template. Each binary node (white circles) is partitioned based on a single feature, and each branch ends in a terminal node, where the prediction of the class is provided. The different colors of the branches represent each of the trees in the forest. The final prediction for a test set is obtained by combining with a majority vote the predictions of all single trees.

RF (see Figure 1 for an illustration) is a collection or ensemble of Classification and Regression Trees (CART) (Breiman et al., 1984) trained on datasets of the same size as training set, called *bootstraps*, created from a random resampling on the training set itself. Once a tree is constructed, a set of bootstraps, which do not include any particular record from the original dataset [*out-of-bag* (OOB) samples], is used as test set. The error rate of the classification of all the test sets is the OOB estimate of the generalization error. Breiman (1996) showed by empirical evidence that, for the bagged classifiers, the OOB error is accurate as using a test set of the same size as the training set. Thus, using the OOB estimate removes the need for a separate test set. To classify new input data, each individual CART tree (colored branches in Figure 1) votes for one class and the forest predicts the class that obtains the plurality of votes.

RF follows specific rules for tree growing, tree combination, self-testing and post-processing, it is robust to overfitting and it is considered more stable in the presence of outliers and in very high dimensional parameter spaces than other machine learning algorithms (Caruana and Niculescu-Mizil, 2006; Menze et al., 2009). The concept of variable importance is an implicit feature selection performed by RF with a random subspace methodology, and it is assessed by the Gini impurity criterion index (Ceriani and Verme, 2012). The Gini index is a measure of prediction power of variables in regression or classification, based on the principle of impurity reduction (Strobl et al., 2007); it is non-parametric and therefore does not rely on data belonging to a particular type of distribution. For a binary split (white circles in Figure 1), the Gini index of a node *n* is calculated as follows:

$$\begin{array}{l} Gini\left(n\right)=1-\sum_{j=1}^{2}{\left(p_{j}\right)}^{2} \end{array}$$

where *p*_*j*_ is the relative frequency of class *j* in the node *n*.

For splitting a binary node in the best way, the improvement in the Gini index should be maximized. In other words, a low Gini (i.e., a greater decrease in Gini) means that a particular predictor feature plays a greater role in partitioning the data into the two classes. Thus, the Gini index can be used to rank the importance of features for a classification problem.

## Methods

**Figure 2:**
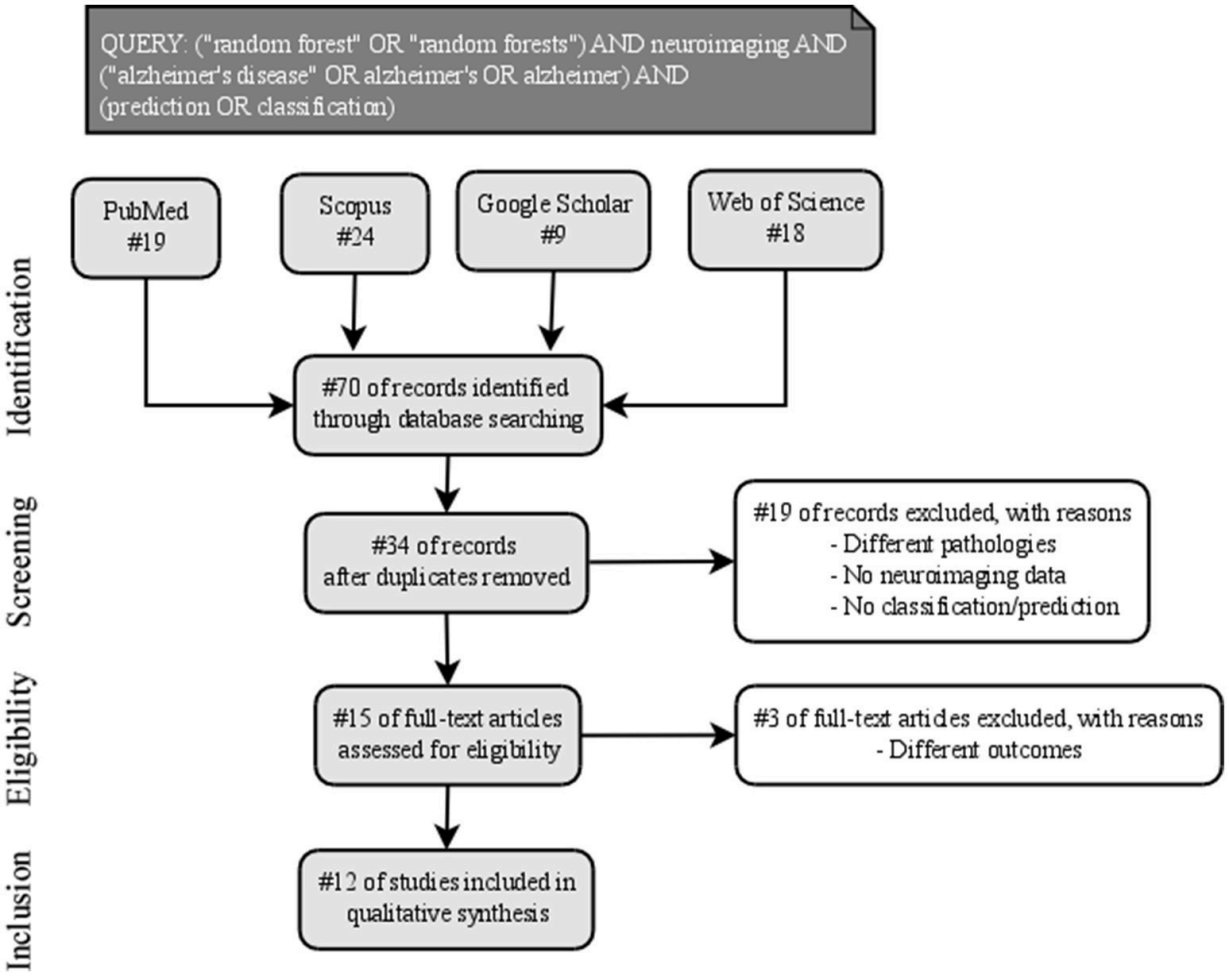
PRISMA workflow of the identification, screening, eligibility, and inclusion of the studies in the systematic review.

For the present systematic review, we followed the Preferred Reporting Items for Systematic Reviews and Meta-Analysis (PRISMA) guidelines (Liberati et al., 2009; Moher et al., 2009). The statement consists of a checklist of recommended items to be reported and a four-step flow diagram (Figure 2).

Published titles and abstracts in the English language from the first of January 2007 to the first of May 2017 were searched systematically across the following databases: PubMed, Scopus, Google Scholar, and Web of Science. The search terms were concatenated in an advanced query using boolean operators as follows: *(“random forest” OR “random forests”) AND neuroimaging AND (“alzheimer's disease” OR alzheimer's OR alzheimer) AND (prediction OR classification)*. After the initial web search, duplicate items among databases were removed.

During the screening phase, to be assessed for eligibility, studies were required to: (1) investigate a cohort of AD in cross-sectional case-control or longitudinal design, (2) analyze neuroimaging data, (3) apply RF algorithm as Machine Learning technique for the classification of AD patients.

To reduce a risk of bias, two authors (A.S. and A.C.) independently screened paper abstracts and titles, and analyzed the full papers that met the inclusion criteria, as suggested by the PRISMA guidelines. The reference lists of examined full-text papers were also scrutinized for additional relevant publications.

Data extracted from the studies—finally included in the qualitative synthesis—were: (1) sample diagnosis, (2) sample size and mean age, (3) neuroimaging acquisition type, (4) features of interest, (5) RF classification parameters, (6) classification performance validation, and (7) selected findings in terms of classification performance.

## Results

### Study selection

Figure 2 reported the four phases—identification, screening, eligibility and inclusion—of the process for the selection of the studies in this review. Nineteen records were excluded after the initial screening of title and abstract and three more records were removed after the full-text assessment, following the inclusion criteria. Finally, 12 studies were included in qualitative synthesis.

### Study characteristics

Data extracted from the studies were summarized in Table 1. In particular, we reported those characteristics that are related to the highest performance reached by RF in each study. Regarding the cohort diagnosis, two works (Tripoliti et al., 2007; Lebedev et al., 2014) investigated Alzheimer's patients (AD) and healthy controls (HC), four works (Cabral et al., 2013; Sivapriya et al., 2015; Maggipinto et al., 2017; Son et al., 2017) had AD, HC, and MCI, two studies (Gray et al., 2013; Moradi et al., 2015) considered AD, HC, stable MCI (sMCI), and progressive MCI (pMCI, converted to AD), two had sMCI and pMCI (Wang et al., 2016; Ardekani et al., 2017), one had HC and MCI (Lebedeva et al., 2017) and one (Oppedal et al., 2015) had AD, HC, and Lewy-body dementia (LBD) patients.

**Table 1:** Characteristics of each of the twelve studies included in the systematic review.

**Study**	**Cohort**	**Nr. of subjects (mean age, nr. of females)**	**Neuroimaging acquisition**	**Features of interest**	**Classification parameters**	**Performance validation**	**Results**
Tripoliti et al., 2007	AD HC	12 (77.2, 7) 14 (74.9, 9)	– 1.5 T MRI – Task-based fMRI	– Demographic data; – Behavioral data; – Head motions parameters; – Volumetric measures; – Activation patterns; – BOLD-derived hemodynamic measures.	– Feature selection based on correlation; – RF with 10 trees.	10-fold cross-validation sensitivity/specificity	AD vs. HC: 98%/98%
Gray et al., 2013	AD sMCI pMCI HC	37 (76.8, 14) 34 (75.7, 12) 41 (76.1, 12) 35 (74.5, 12)	– 1.5 T MRI – FDG-PET	– Volumetric measures; – FDG-PET voxel intensities whole-brain; – CSF-derived measures; – Genetic information.	– RF with 5,000 trees.	Stratified repeated random sampling accuracy on a separate test set	AD vs. HC: 89% MCI vs. HC: 74.6% sMCI vs. pMCI: 58.4%
Cabral et al., 2013	AD MCI HC	59 (78.2, 25) 59 (77.7, 19) 59 (77.4, 21)	– FDG-PET	– FDG-PET voxel intensities;	– Feature selection with Mutual Information criterion; – Decomposition by the one-vs.-all scheme; – Aggregation scheme with voting strategy (MAX); – RF with 100 trees.	Repeated 10-fold cross-validation accuracy	AD vs. MCI vs. HC: 64.63%
Lebedev et al., 2014	AD HC	185 (75.2, 92) 225 (75.95, 110)	– 1.5 T MRI	– Non-cortical volumes; – Cortical thickness; Jacobian maps; – Sulcal depth.	– Recursive feature elimination with Gini index; – RF with 1,000 trees.	Overall accuracy on a separate test set	AD vs. HC: 90.3%
Moradi et al., 2015	AD sMCI pMCI HC	200(55-91, 97) 100 (57-89, 34) 164 (77-89, 67) 231 (59-90, 112)	– 1.5 T MRI	– GM density values; – Age; – Neuropsychological scores.	– Feature selection with regularized logistic regression framework	10-fold cross-validation accuracy	sMCI vs. pMCI: 82%
Oppedal et al., 2015	AD LBD HC	57 (N.A.) 16 (N.A.) 36 (N.A.)	– 1.0/1.5 T MRI– FLAIR	– Local binary pattern (LBP); – Image contrast measure (C).	– RF with 10 trees.	10-fold nested cross-validation accuracy	AD vs. LBD vs. HC: 87% AD+LBD vs. HC: 98% AD vs. LBD: 74%
Sivapriya et al., 2015	AD MCI HC	140 (N.A.) 450 (N.A.) 280 (N.A.)	– MRI – FDG-PET	– Volumetric measures; – FDG-PET uptake ROI-based; – Neuropsychological scores.	– Feature selection with particle swarm optimization approach coupled with the Merit Merge technique (CPEMM); – RF with 100 to 1,000 trees.	5-fold cross-validation accuracy	AD vs. MCI vs. HC: 96.3%
Wang et al., 2016	sMCI pMCI	65 (72.2, 26) 64 (72.5, 29)	– 1.5 T MRI – florbetapir-PET – FDG-PET	– Morphological measures; – florbetapir-PET uptake whole-brain; – FDG-PET uptake whole-brain.	– RF with 500 trees.	Leave-one-out cross-validation accuracy	sMCI vs. pMCI: 73.64%
Ardekani et al., 2017	sMCI pMCI	78 (74.75, 24) 86 (74.10, 31)	– 1.5 T MRI	– Hippocampal volumetric integrity; – Neuropsychological scores.	– Feature selection with Gini index; – RF with 5,000 trees.	OOB estimation of classification accuracy	sMCI vs. pMCI: 82.3%
Lebedeva et al., 2017	MCI HC	32 (78.1, 22) 40 (76.4, 29)	– 1.5/3 T MRI	– Cortical thickness; – Subcortical volumes. – MMSE	– Feature selection with Gini index; – RF with 5,000 trees.	OOB estimation of classification accuracy	MCI vs. HC: 81.3%
Maggipinto et al., 2017	AD MCI HC	50 (N.A.) 50 (N.A.) 50 (N.A.)	– DTI	– TBSS FA voxels;	– Feature selection with the Wilcoxon rank sum test and ReliefF algorithm; – RF with 300 trees.	Repeated 5-fold cross-validation accuracy	AD vs. HC: 87% MCI vs. HC: 81%
Son et al., 2017	AD MCI HC	30 (74, 18) 40 (74.3, 21) 35 (76.06, 23)	– 3 T MRI – rs-fMRI	– Subcortical volumes; – Eigenvector centrality of functional networks ROI-based.	N.A.	Repeated leave-one-out cross-validation accuracy	AD vs. MCI vs. HC: 53.33%

*Data are related to the highest performance reached by random forest. AD, Alzheimer's disease; HC, healthy controls; MCI, Mild cognitive impairment; cMCI, converter MCI; pMCI, progressive MCI; LBD, Lewy-body dementia; MRI, Magnetic resonance imaging; fMRI, functional MRI; rs-fMRI, resting state fMRI; PET, positron emission tomography; FDT-PET, fluorodeoxyglucose PET; DTI, Diffusion tensor imaging; GM, Gray matter; ROI, Region of interest; MMSE, Mini mental state examination; TBSS, Tract-based spatial statistics; OOB, out-of-bag; N.A., not applicable*.

All studies, except two (Cabral et al., 2013; Maggipinto et al., 2017), which used FDG-PET and DTI acquisition respectively, investigated structural MRI data alone (Lebedev et al., 2014; Moradi et al., 2015; Ardekani et al., 2017; Lebedeva et al., 2017) or in combination with features extracted from other modalities, that is FDG-PET (Gray et al., 2013; Sivapriya et al., 2015), florbetapir-PET (Wang et al., 2016), FLAIR (Oppedal et al., 2015) and fMRI (Tripoliti et al., 2007; Son et al., 2017).

**Figure 3:**
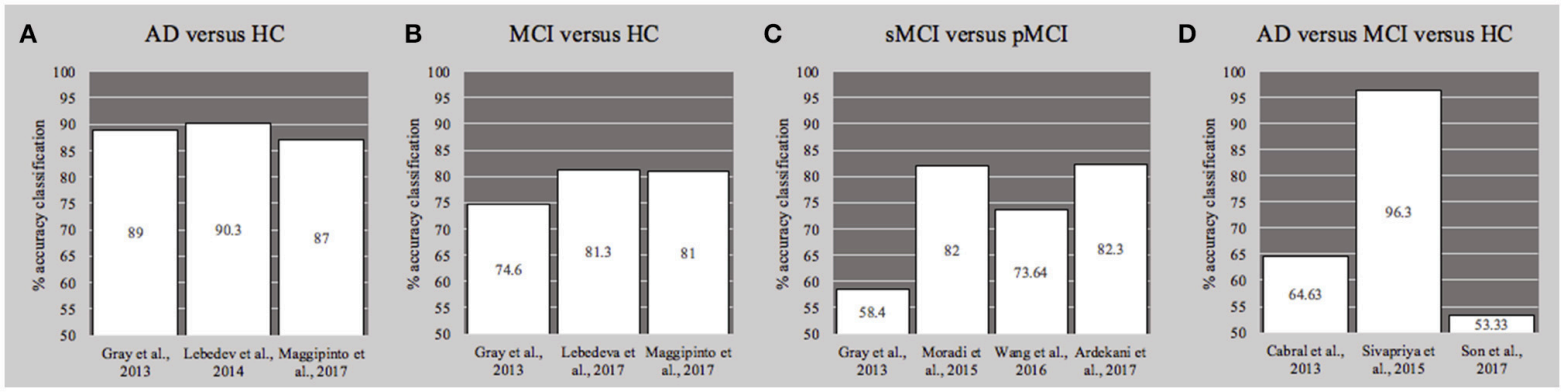
Histograms of the overall accuracy (%) reached by the studies—where applicable—for the binary classifiers **(A)** AD vs. HC, **(B)** MCI vs. HC and **(C)** sMCI vs. pMCI, and for the ternary problem **(D)** AD vs. MC vs. HC. See also Table 1. AD, Alzheimer's disease; HC, healthy controls; MCI, Mild cognitive impairment; cMCI, converter MCI; pMCI, progressive.

Eight works (Tripoliti et al., 2007; Cabral et al., 2013; Lebedev et al., 2014; Moradi et al., 2015; Sivapriya et al., 2015; Ardekani et al., 2017; Lebedeva et al., 2017; Maggipinto et al., 2017) applied feature selection/elimination for reducing the dimension of the variables space. The number of trees used in the RF was not specified in two cases (Moradi et al., 2015; Son et al., 2017). Finally, we reported in the column Results of Table 1 the—highest—overall accuracies of binary or ternary classifiers reached by each study, except for the one (Tripoliti et al., 2007) that provided only sensitivity and specificity. Figure 3 presented a comparison—where applicable—of accuracies obtained by the studies for the binary models AD vs. HC (Figure 3A, with a mean of 88.8%), MCI vs. HC (Figure 3B, with a mean of 79%), sMCI vs. pMCI (Figure 3C, with a mean of 74%), and the multi-class problem AD vs. HC vs. MCI (Figure 3D, with a mean of 71.42%)

More details about individual works, such as, the results obtained with other algorithms or other subsets of features, could be found in the next section.

### Results of individual studies

#### Tripoliti et al. (2007)

Tripoliti et al. (2007) conducted a study on 41 subjects, divided into three groups: 12 subjects were AD patients (mean age 77.2, 7 females), from very mild to mild following the Clinical Dementia Rating (CDR = 0.5/1), 14 subjects were healthy young controls (mean age 21.1, 9 females, CDR = 0) and 14 were healthy elderly subjects (mean age 74.9, 9 females, CDR = 0).

The cohort underwent a visual fMRI finger tapping task. Raw structural and functional images were preprocessed for correction of motion artifacts, registered and normalized.

Demographic and behavioral data were grouped with the features extracted from the data preprocessing phase: (i) head motions parameters; (ii) volumetric measures, i.e., volumes obtained from the segmentation of gray matter (GM), WM and CSF; (iii) activation patterns, consisting in several measures derived from the activated voxels and clusters; (iv) hemodynamic measures extracted from the BOLD responses, such as, the amplitude of venous volume or of vascular signal. Authors applied a feature selection on this dataset for reducing the dimensionality by removing highly correlated variables. Selected features were used for training a RF classifier with 10 trees, and the performance was assessed using 10-fold cross-validation accuracy. Two separated datasets were evaluated: the first consisted of AD patients and both young and old healthy subjects, while the second consisted of AD and only old controls. Sensitivity and specificity of the two binary classifiers were ranging from 94 to 98%, depending of the subset of selected features. The highest values were obtained on the dataset that included AD and old controls, with a 98% of both sensitivity and specificity.

#### Gray et al. (2013)

Gray et al. (2013) selected a cohort of 147 subjects from the ADNI database, consisting of 37 AD patients (mean age 76.8, 14 females, CDR = 0.5/1), 75 MCI patients divided into 34 stable MCI (sMCI, mean age 75.7, 12 females, CDR = 0.5) and 41 subjects progressed to AD (pMCI, mean age 76.1, 12 females, CDR = 0.5), and 35 HC (HC, mean age 74.5, 12 females, CDR = 0). All subjects underwent morphological 1.5T MRI, FDG-PET, and CSF analysis at the baseline and authors used already pre-processed data by ADNI. In particular, structural MRI and FDG-PET images were motion-corrected, examined for major artifacts and registered to the standard space MNI. Eighty-three volumetric region-based features were extracted from MRI, while signal intensities of 239,304 voxels were obtained from FDG-PET. Biological features were CSF-derived measures of Aβ, tau, and ptau. Furthermore, a categorical variable describing the ApoE genotype was used as genetic feature.

Three different binary datasets were used for the RF classification: AD vs. HC, MCI vs. HC, sMCI vs. pMCI. The performance of each classifiers was evaluated with a stratified repeated random sampling approach, where, in each of the 100 runs, the dataset was divided into training (75%) and test set (25%). Accuracy on the test set was then calculated as the mean of all the 100 repetitions. The RF models were trained with 5,000 trees on the feature data from each of the four modalities independently and the feature importance ranking was extracted. As further analysis, authors measured the similarity between pairs of examples from the RF classifiers and applied a Manifold learning approach on data from single-modality and on combined/concatenated features (multi-modality).

Although the single-modality classification results were comparable between the original dataset and the embedded feature one, the latter presented the best performances as following: 86.4% for the AD vs. HC with the FDG-PET data, 73.8% for MCI vs. HC with the genetic data, 58.4% for the sMCI vs. pMCI with MRI data. A slight increase of the accuracy was obtained with the multi-modality classification for AD vs. HC (+2.6%) and for MCI vs. HC (+0.8%), while for pMCI vs. sMCI there was a small decrease (−0.4%).

#### Cabral et al. (2013)

Cabral et al. (2013) collected 177 subjects from the ADNI database, divided into three balanced groups: AD patients (mean age 78.2, 25 females, CDR > 0.5), MCI patients (MCI, mean age 77.7, 19 females, CDR = 0.5) and HC (mean age 77.4, 21 females, CDR = 0). Authors analyzed FDG-PET data, acquired 24 months after the first visit and already pre-processed by ADNI. In particular, they used the voxel intensities (VI) as features of interest, for a total of 309,881 variables. The original dataset was decomposed by using the one-vs.-all scheme, resulting into three subsets: AD vs. ALL, MCI vs. ALL, HC vs. ALL. The Mutual Information criterion was used for extracting the optimal features with the highest ranking value, separately for each pairwise problem. The selected features were then used for training three binary RF models with 100 trees. As aggregation scheme for the ternary problem, the voting strategy (MAX) was applied. The classification performance was then assessed by the 10-fold cross-validation accuracy, repeated 5 times with fold randomization. The ternary RF classifier provided a multiclass accuracy of 64.63%. It must be addressed that the authors applied other two algorithms, linear and RBF SVM, obtaining, respectively, an accuracy of 66.33% and 66.78%.

#### Lebedev et al. (2014)

The study of Lebedev et al. (2014) was based on a cohort of 575 subjects from ADNI database, divided into three main groups: 185 AD (mean age 75.2, 92 females, CDR = 1), 165 patients with MCI (mean age 75.46, 62 females, CDR = 0.5) of which 149 progressed to AD within 4 years, and 225 HC (mean age 75.95, 110 females, CDR = 0). The MCI group was split into six subgroups according to the month of MCI-to-AD conversion (6th-, 12th-, 18th-, 24th-, 36+th-month converters and non-converters).

The features of interest were extracted from 1.5 T MRI images using a surface-based cortex reconstruction and volumetric segmentation. In particular, (i) non-cortical volumes, (ii) cortical thickness (CTH), (iii) Jacobian maps and (iv) sulcal depth were measured for each subject. The ability of these parameters in distinguishing AD from HC, was assessed individually and with a combination of measurements of CTH and non-cortical volumes.

The feature importance was assessed with the intrinsic characteristic of RF consisting of the recursive feature elimination (RFE) with the Gini index as criterion and 10,000 trees. The performance of models—with and without RFE—was evaluated as the overall accuracy on a separate test set with 35 AD and 75 HC. Findings revealed that the highest accuracy (90.3%) for the classifier AD vs. HC was obtained with the RFE on the combined dataset with thickness and non-cortical volumes. An increase of 0.7% was found in this accuracy when authors combined all models by a majority vote approach. The majority vote method resulted to have also the best ability to predict MCI-to-AD conversion 2 years before actual dementia onset with sensitivity/specificity of 76.6/75%. As further analysis, authors found that the adding of ApoE genotype and demographics data did not improve the overall accuracy in distinguishing AD from HC, while it showed an increase of sensitivity/specificity (83.3/81.3%) in the prediction of MCI conversion.

#### Moradi et al. (2015)

Moradi et al. (2015) obtained baseline data for their analysis from the ADNI database and they selected 825 subjects grouped as: 200 AD patients (age range 55–91, 97 females), 100 stable MCI (sMCI, age range 57–89, 34 females), 164 MCI progressed to AD within 3 years from the baseline (pMCI, age range 77–89, 67 females) and 231 HC (age range 59–90, 112 females). Another group of 100 unknown MCI (uMCI, age range 54–90, 81 females) diagnosed as MCI at the baseline but with missing diagnosis at 36 months follow-up was also considered. For integrating the unlabeled group of uMCI into the training set and assigning them to the pMCI or sMCI class, the authors used a low density separation (LDS) approach for semi-supervised learning.

All subjects underwent 1.5 T MRI acquisition and the T1-w scans were preprocessed following the voxel-based morphometry approach. In particular, T1-w images were corrected, spatially normalized and segmented into GM, WM, and CSF. The GM maps were then further processed for extracting 29,852 GM density values—for each subject—used as MRI features for the classification task.

The high number of GM voxels was reduced with a feature selection approach consisted in the regularized logistic regression framework applied only on the dataset with AD and HC subjects. The selected variables were then aggregated with age and cognitive measurements and used for building the RF classifier for predicting AD in MCI patients, i.e., sMCI vs. pMCI.

The RF model performance was evaluated as the mean accuracy calculated by 10-fold cross-validation. The highest accuracy in distinguishing the MCI-to-AD conversion reached almost the 82% when the concatenated measures—age, cognitive, and voxel—and the combination of LDS and RF were considered. The importance analysis of MRI features, age, and cognitive measurements calculated by RF classifier revealed that the first three most predictive variables were: MRI voxels, the Rey's Auditory Verbal Learning Test (RAVLT) and the Alzheimer's Disease Assessment Scale—cognitive subtest 11 (ADAS-cog total-11).

#### Oppedal et al. (2015)

In Oppedal et al. (2015), a total of 73 mild dementia subjects, divided into 57 AD patients and 16 LBD patients, together with 36 HC were investigated. The cohort MRIs were acquired in different research centers with 1.0/1.5 T scanners and FLAIR images were also obtained. T1-w images were corrected, registered and segmented for extracting the white matter (WM) tissue. From the pre-processed FLAIR images, the WM lesions (WML) maps were automatically created. In a second phase of the study, authors applied the local binary pattern (LBP) approach as a texture descriptor on both T1 and FLAIR images and their derived WM and WML maps as ROIs. For enhancing the discriminative power of LBP, an image contrast measure (C) was added as variable for every voxel in the specified ROI. The total number of features for each subject was 48, resulting from the combination of LBP and C values in each ROI.

Feature selection and classification were performed with a RF classifier with 10 trees and the 10-fold nested cross validation accuracy was used as the performance metric. In particular, three RF models were built: (i) a ternary problem HC vs. AD vs. LBD, (ii) a binary classifier HC vs. AD+LBD and (iii) another binary model AD vs. LBD.

For the ternary problem—HC vs. AD vs. LBD—the best accuracy (87%) was reached when the classifier was trained on the texture features extracted from the T1 images in the WML masks (T1WML). Results of the model HC vs. AD+LBD revealed that the highest accuracy (98%) was obtained also when only T1WML variables were considered. On the contrary, for distinguishing AD from LBD with the maximum accuracy (74%) the texture features should be extracted from the T1 in the WM ROI.

#### Sivapriya et al. (2015)

Four datasets from the ADNI database were used by Sivapriya et al. (2015) and three different groups of subjects were selected: AD, MCI, and HC. The number of subjects in each dataset varied according to the features considered: (i) Neuropsychological dataset (150 AD, 400 MCI, 200 HC), (ii) Neuroimaging dataset (250 AD, 200 MCI, 250 HC), (iii) Baseline combined data with both neuropsychological and neuroimaging measures (140 AD, 450 MCI, 280 HC), and (iv) combined dataset (150 AD, 400 MCI, 200 HC). Some of the neuropsychological tests used were the Clinical dementia ratio-SB, the ADAS, the RAVLT, and the MOCA. Authors used already pre-processed MRI data by ADNI for their study, in particular neuroimaging measures extracted from T1-w and FDG-PET images, consisting in volumes and average PIB SUVR of several regions of interest (ROIs).

The feature selection and classification task was composed by three main phases in which RF performance was evaluated together with other ensemble algorithms—Naïve Bayes, J48 and SVM. Each classifier was trained with each of the four datasets, after that they were dimensionally reduced with a particle swarm optimization approach coupled with the Merit Merge technique (CPEMM). The performance of the classification models was evaluated with the 5-fold cross-validation accuracy of the ternary problem AD vs. MCI vs. HC. RF—implemented with 100 to 1,000 trees—showed its best multi-class accuracy (96.3%) when it was trained on the baseline combined dataset and the same result was obtained with the CPEMM feature selection methodology. It must be addressed that RF reached comparable performance of the other classification algorithms, except for SVM that presented the lowest accuracies in the delineation of dementia.

#### Wang et al. (2016)

The study of Wang et al. (2016) included 129 subject with MCI (CDR = 0.5) from the ADNI database. The cohort was divided into 65 stable MCI (sMCI, mean age 72.2, 26 females) and 64 progressive MCI (pMCI, mean age 72.5, 29 female), who converted to AD within 3 years from the baseline. All subject underwent the acquisition of 1.5 T MRI, florbetapir-PET and FDG-PET. Authors analyzed already pre-processed neuroimaging data by ADNI, separately grouped according to the modality of acquisition, i.e., features extracted from the T1-w images and the uptake of florbetapir and FDG. A dataset with a combination of these multimodal measures was also evaluated. Three classification algorithms—partial least square (PLS, informed and agnostic), linear SVM and RF (500 trees)—were trained on these four different datasets. Their ability in distinguishing sMCI from pMCI was assessed with the leave-one-out cross-validation accuracy. RF showed the best accuracy (73.64%) when it was trained on the combined multi-modal features dataset. A comparable result (76.74%) on the same dataset was reached by SVM. On the contrary, informed PLS generally outperformed both RF and SVM especially when the three neuroimaging modalities are fused (81.4% of accuracy).

#### Ardekani et al. (2017)

Ardekani et al. (2017) applied their classification task on a cohort of 164 MCI (CDR = 0.5) patients from the ADNI database, divided into 78 stable MCI (sMCI, mean age 74.75, 24 females) and 86 MCI converted to AD within 3 years from the baseline (pMCI, mean age 74.10, 31 females). All selected subjects underwent two 1.5 T MRI acquisitions, at the baseline and at ~1 year later. Neuropsychiatric scores of these two time points were also considered in the analysis. T1-w images—without any pre-processing—were used for calculating the hippocampal volumetric integrity (HVI), defined as the fraction of volume of a region that is expected to surround the hippocampus in a normal brain that is occupied by tissue (rather than CSF). The HVI is measured—separately for each hemisphere—as the area under the histogram curve for voxel values above a CSF intensity threshold. The HVI measures and the neuropsychiatric scores were merged for a total of 16 features for each subject, including their average rate of change between the baseline and the 1-year follow-up.

Several RF models (5,000 trees) were trained on different feature subsets and their performance were evaluated with the OOB estimation of classification accuracy. The mean reduction of Gini impurity index was used for the assessment of the variable importance.

The highest accuracy (82.3%) in distinguishing between sMCI and pMCI was reached when the combination of neuroimaging and neuropsychiatric features was considered as training set. The classifiers built only on the baseline measures or only on HVI values showed indeed poor performance. The variable ranking of the 16 features revealed that—according to the impurity criterion—ADAS cognitive test was the most important one, followed by the rate of change of the right HVI.

#### Lebedeva et al. (2017)

The work of Lebedeva et al. (2017), was aimed at predicting MCI and dementia in late-life depression (LLD) patients 1 year prior to the diagnosis. The analysis was conducted on a cohort of 32 patients (MCI-DEM, mean age 78.1, 22 females) including 21 MCI and 8 AD, and a group of 40 age—sex—matched HC (mean age 76.4, 29 females) from the PRODE prospective multicenter study (Borza et al., 2015). All subjects underwent 1.5/3 T MRI acquisition at the baseline and after 1 year. T1-w images were pre-processed for extracting CTH and subcortical volumes (SV) with a standard pipeline, for a total of 148 features. Clinical and neuropsychological assessment was performed for each subject at both time points.

Several RF models (5,000 trees) were built for classifying MCI-DEM or MCI vs. HC at 1-year follow—up, by varying the feature space, i.e., separated CTH and SV variables, a combination of CTH and SV, and with/without the addition of demographic and clinical data. The OOB overall accuracy was assessed as performance metric. The model for discriminating MCI-DEM from HC reached the best result (81.3% of OOB overall accuracy) when the CTH, SV, and MMSE values were combined together. The accuracy resulted to be higher (90.1%) in the model of MCI (excluding AD patients) vs. HC with SV and MMSE as training features. The variable importance ranking—measured with the Gini criterion—showed that, in every RF models, the most relevant features were the right ventral diencephalon, the middle anterior corpus callosum and the right hippocampus.

As further analysis, authors used their PRODE cohort (MCI-DEM and HC) as test set for the RF model previously built by Lebedev et al. (2014) on AD and HC from ADNI database. The accuracy was better (67%) when only SV measures were used than when SV and CTH were combined (57.5%).

#### Maggipinto et al. (2017)

The cohort investigated by Maggipinto et al. (2017) was obtained from ADNI database and it consisted of 150 subjects divided into three groups: 50 AD, 50 MCI, and 50 HC with an age range from 55 to 90. Diffusion-weighted scans acquired with a 3 T scanner was used for this machine learning study, randomly selected from the baseline and follow-up visit. DTIs were pre-processed for correction of movement artifacts and eddy currents with a standard pipeline. A diffusion tensor was fitted for each subject and fractional anisotropy (FA) and mean diffusion (MD) maps were extracted. The FA and MD maps were then used as input for a tract-based spatial statistics (TBSS) analysis, which—for each subject —produced ~120,000 voxels for each diffusion metric.

In a first phase, authors assessed the importance value of the voxels in discriminating AD from HC with two different feature selection methods: the Wilcoxon rank sum test and the ReliefF algorithm, which were used both within a non-nested and nested approach. For the classification task, fifteen subsets were then created by selecting an increasing number—from 50 to 3,000—of most discriminating voxels, ordered by decreasing importance. RF models were trained with 300 trees on each of these feature subspaces and their performance was evaluated with a repeated (100 runs) 5-fold cross-validation accuracy.

The models built on the FA features selected with the non-nested approach showed the highest accuracies in both binary problems, AD vs. HC (87%) and MCI vs. HC (81%). The non-nested variable selection resulted to produce better results than the nested one also when MD voxels were used for training the classifiers (83% for AD vs. MCI and 79% for MCI vs. HC).

#### Son et al (2017)

A sample of 105 subjects was selected by Son et al. (2017) from the ADNI database. The cohort was divided into three age—sex—matched groups: 30 AD (mean age 74, 18 females), 40 MCI (mean age 74.3, 21 females) and 35 HC (mean age 76.06, 23 females). All participants underwent 3 T acquisition of T1-w images and resting state functional MRI (rs-fMRI). Structural scans were pre-processed for correcting movement artifacts and smoothed, and then they were segmented into WM, GM, and CSF. The volumes of 10 subcortical regions were calculated as measure of atrophy. The rs-fMRI images were pre-processed and registered onto the T1-w and aligned to the MNI standard space. Given a set of ROIs from the AAL atlas as nodes, the functional networks were constructed by defying the edges as correlation values between nodes. Authors quantified the connectivity of the functional networks within the 10 subcortical regions with the eigenvector centrality measure among AD and HC, MCI and HC, and AD and MCI.

The ternary problem, AD vs. MCI vs. HC, was evaluated by training a RF classifier with the SV and the eigenvector centrality measures as features. The multi-class accuracy of the RF model was assessed with a repeated (105 runs) leave-one-out cross-validation approach. Authors reached a poor performance (accuracy: 53.33%) in distinguishing among AD, MCI, and HC subjects. However, they identified distinctive regional atrophy and functional connectivity patterns characterizing each binary problem AD vs. HC (thalamus, putamen and hippocampus bilaterally and left amygdala), MCI vs. HC (left putamen and right hippocampus), and MCI vs. AD (bilateral hippocampus and right amygdala).

## Discussion

RF has been successfully applied in many scientific realms such as, the bioinformatics, proteomics, and genetics (Menze et al., 2009; Calle et al., 2011; Chen et al., 2011), but it was less applied on neuroimaging data for the prediction of the Azheimer's disease. The present paper is the first, to our knowledge, that systematically analyzed the literature of the last 10 years on the use of the RF algorithm on neuroimaging data for the early diagnosis of AD. In this review, we summarized the characteristics of twelve works (Tripoliti et al., 2007; Cabral et al., 2013; Gray et al., 2013; Lebedev et al., 2014; Moradi et al., 2015; Oppedal et al., 2015; Sivapriya et al., 2015; Wang et al., 2016; Ardekani et al., 2017; Lebedeva et al., 2017; Maggipinto et al., 2017; Son et al., 2017) by focusing our attention on performance reached by their algorithms.

A direct comparison of the results of the selected works is influenced by several factors, such as, the different sample sizes, neuroimaging modalities, and different methods for the feature selection. However, we found several points in common among papers, such as, similar performance validation approaches, as well as a general trend showing that the classification based on a combination of features extracted from different categories improved the ability in predicting AD. Another important common aspect of the selected articles is the use of data from the ADNI database. Indeed, 10 works (Cabral et al., 2013; Gray et al., 2013; Lebedev et al., 2014; Moradi et al., 2015; Sivapriya et al., 2015; Wang et al., 2016; Ardekani et al., 2017; Lebedeva et al., 2017; Maggipinto et al., 2017; Son et al., 2017) applied their methodologies on ADNI cohorts.

The best accuracies—around 90%—for the binary problem AD vs. HC, were observed when the RF classifiers were trained on high-dimensional and multi-modality data (Tripoliti et al., 2007; Cabral et al., 2013; Gray et al., 2013; Lebedev et al., 2014). Superior performance of these models can be explained by the ability of RF to detect less extensive changes in the variables, which could be not revealed by others algorithms. Moreover, Moradi et al. (2015) showed that RF was more immune to the data type thanks to its capability to handle discrete data and to apply an efficient discretization algorithm on continuous data type before the learning step.

The binary models for distinguishing MCI from HC and stable MCI from progressive MCI showed lower accuracies, around 82%, although it was similarly improved by multi-modal data classification (Figure 3). In particular, the inclusion of age as well as cognitive measurements (MMSE and ADAS-cog), in the space of features, significantly increased the classification of MCI vs. HC (Gray et al., 2013; Lebedeva et al., 2017; Maggipinto et al., 2017) and the AD conversion prediction in MCI patients (Moradi et al., 2015; Wang et al., 2016; Ardekani et al., 2017). On the contrary, for the conundrum between sMCI vs. pMCI, Gray et al. (2013) found that the accuracy reached on multi-modality classification is not significantly different from that obtained with MRI information alone. Interestingly, authors suggested that the lack of improvement in distinguishing the progression to AD, could be overcame by incorporating longitudinal information, as indeed Ardekani et al. (2017) demonstrated afterwards by considering the rate of change of variables.

Three works (Cabral et al., 2013; Sivapriya et al., 2015; Son et al., 2017) investigated the ternary problem: AD vs. MCI vs. HC, but only the work of Sivapriya et al. (2015) reached a reliable accuracy of 96.3%. The low performance of the other two studies—64.63% of Cabral et al. (2013) and 53.33% of Son et al. (2017)—might be due to the heterogeneous pattern of brain changes across the three groups and the inability of RF to model the too large variability in the stages of pathological process. Thus, although RF can be naturally extended to multi-class problems, the AD vs. MCI vs. HC ternary model could not be still translated into a real-world clinical scenario.

Another interesting observation was that, both in binary and ternary problems, feature selection based on the Gini index, improved the overall performance and this is true also for the works in which only a neuroimaging modality was used (Lebedev et al., 2014; Ardekani et al., 2017; Lebedeva et al., 2017; Maggipinto et al., 2017). Other kinds of feature selection and extraction, applied prior to the RF classification, showed also an improvement in the overall accuracies (Tripoliti et al., 2007; Cabral et al., 2013; Moradi et al., 2015; Oppedal et al., 2015; Sivapriya et al., 2015; Wang et al., 2016; Ardekani et al., 2017; Lebedeva et al., 2017; Maggipinto et al., 2017).

A further interesting characteristic of the RF algorithm in the AD realm was the estimates of the features importance. The ranking of the variables plays an important role because it could assess which of the features contribute most to the prediction by also providing a correspondence to anatomical regions or structures with a biologically plausible connection to pathology (Gray et al., 2013; Lebedev et al., 2014; Moradi et al., 2015; Ardekani et al., 2017).

A limitation of this systematic review concerns the lack of information about the tuning of the RF parameters. In particular, poor information were reported in the selected works about how the number and depth of trees in the forest or the splitting criteria were chosen. Although, this tuning is performed automatically by RF, how external assessment of these parameters (i.e., cross-validation approach) would improve the overall accuracies is still unknown.

Again, what still remains to be assessed is the performance of RF algorithm on multi-site data. As already demonstrated for rs-fMRI datasets from different sites (Abraham et al., 2017; Dansereau et al., 2017), the accuracy and the reliability of the biomarkers extraction could be enhanced by dramatically increasing the cohort size. Moreover, it was shown that classifiers trained on data from multiple sources will likely generalize better to new observations (Dansereau et al., 2017), avoiding the overfitting. Thus, it would be interesting to evaluate how well RF could classify when it is trained on features that are not invariant across sites and how the sample heterogeneity influences its performance.

This systematic review provided, for the first time, a framework for the exploration of the RF algorithm and of its strength in predicting AD when high-dimensional and multi-modal neuroimaging data are combined with demographics, genetic and cognitive scores. Indeed, as recently stated by Rathore et al. (2017), no single neuroimaging modality is enough to reach optimal accuracy for automatic AD prediction, but only through the combination of different methodologies, the classification task could be effectively translated into the clinical realm. Our work supported the idea that there is some complementary information between modalities and that this knowledge can be successfully explored with a combination of classifiers rather than a single one. The RF, as a bagging ensemble model, provided promising results, but with possible limitations. Thus, given the high accuracies reached by RF in the classification of dementia, we aimed at encouraging further studies, especially for comparing and integrating this algorithm with other machine learning approaches, such as, the deep learning, which recently showed its potentiality in the investigation of neuroimaging correlates (Shen et al., 2017; Vieira et al., 2017). In the future, the aggregation of multi-approaches (RF, Deep-learning and SVM), multimodal (MRI, DTI, PET) and multi-sites data would drastically increase our ability to extract reliable biomarkers of neurodegenerative diseases.

## Author contributions

AS: Research project: Conception, Organization, and Execution. Statistical Analysis: Design, Execution, Review, and Critique. Manuscript: Writing of the first draft, Review, and Critique. AC: Research project: Conception, Organization and Execution. Manuscript: Review and Critique. AQ: Research project: Organization and Execution. Manuscript: Review and Critique.

### Conflict of interest statement

The authors declare that the research was conducted in the absence of any commercial or financial relationships that could be construed as a potential conflict of interest.
